# Mass Spectrometry-Based Lipidomics of Brown Adipose Tissue and Plasma of New-Born Lambs Subjected to Short-Term Cold Exposure

**DOI:** 10.3390/ani12202762

**Published:** 2022-10-14

**Authors:** Andrea Graña-Baumgartner, Venkata S. R. Dukkipati, Patrick J. Biggs, Paul R. Kenyon, Hugh T. Blair, Nicolás López-Villalobos, Alastair B. Ross

**Affiliations:** 1 School of Agriculture and Environment, Massey University, Private Bag 11 222, Palmerston North 4442, New Zealand; 2 School of Veterinary Science, Massey University, Private Bag 11 222, Palmerston North 4442, New Zealand; 3 School of Natural Sciences, Massey University, Private Bag 11 222, Palmerston North 4442, New Zealand; 4 Proteins and Metabolites, AgResearch Ltd., Lincoln 7674, New Zealand

**Keywords:** lipidomics, mass spectrometry, BAT, thermogenesis, lambs

## Abstract

**Simple Summary:**

This study evaluated the impact of short-term cold exposure (2-days) on new-born lamb brown adipose tissue (BAT) and plasma lipid composition, and identified potential biomarkers to predict BAT activity. Results from the present study propose that short-term cold exposure provokes profound changes in BAT and the plasma lipidome composition of new-born lambs. A significant increase in key lipid classes was observed, where they seem to cooperate as one in order to enhance lipid metabolism via BAT thermogenic activation and adipocyte survival during cold adaptation.

**Abstract:**

During cold exposure, brown adipose tissue (BAT) holds the key mechanism in the generation of heat, thus inducing thermogenic adaptation in response to cooler environmental changes. This process can lead to a major lipidome remodelling in BAT, where the increase in abundance of many lipid classes plays a significant role in the thermogenic mechanisms for heat production. This study aimed to identify different types of lipids, through liquid chromatography–mass spectrometry (LC-MS), in BAT and plasma during a short-term cold challenge (2-days), or not, in new-born lambs. Fifteen new-born Romney lambs were selected randomly and divided into three groups: Group 1 (*n* = 3) with BAT and plasma obtained within 24 h after birth, as a control; Group 2 (*n* = 6) kept indoors for two days at an ambient temperature (20–22 °C) and Group 3 (*n* = 6) kept indoors for two days at a cold temperature (4 °C). Significant differences in lipid composition of many lipid categories (such as glycerolipids, glycerophospholipids, sphingolipids and sterol lipids) were observed in BAT and plasma under cold conditions, compared with ambient conditions. Data obtained from the present study suggest that short-term cold exposure induces profound changes in BAT and plasma lipidome composition of new-born lambs, which may enhance lipid metabolism via BAT thermogenic activation and adipocyte survival during cold adaptation. Further analysis on the roles of these lipid changes, validation of potential biomarkers for BAT activity, such as LPC 18:1 and PC 35:6, should contribute to the improvement of new-born lamb survival. Collectively, these observations help broaden the knowledge on the variations of lipid composition during cold exposure.

## 1. Introduction

Brown adipose tissue (BAT) is the main orchestrator in mammals of thermogenic adaptation in response to a cold challenge [1]. In such environments, BAT metabolism is considerably altered in order to maintain body temperature, boosting its thermogenic capacity by inducing non-shivering thermogenesis [2,3]. There is higher BAT activity in winter [4,5] or after exposure to a cold environment (i.e., 4–5 °C) [6]. The key mechanism that leads to the generation of heat is the action of uncoupled mitochondrial respiration, through uncoupling protein 1 (UCP1) [1]. This protein is able to disrupt adenosine triphosphate synthesis and allows protons to flow across the inner mitochondrial membrane to release energy as heat [7,8]. Further, cold exposure leads to the stimulation of β-adrenergic receptors, which are normally found on the surface of brown adipocytes in BAT [9,10,11]. This stimulus results in the activation of the lysis of triacylglycerols (TAGs) and the subsequent release of long-chain fatty acids [12,13,14], which are the main substrate for UCP1 thermogenic function [7]. The consequent distribution of heat produced through these processes appears to be facilitated by an increased vascularisation [15], to the already highly innervated and vascularised BAT [2,16]. This increase in blood flow also ensures the continuous supply of metabolic substrates needed for thermogenesis [7]. Hence, lipid metabolites that are produced or consumed by BAT could have an impact on the abundance or type of metabolites in plasma, and therefore could be used as predictors for BAT activity. A study by Boon et al. [17] in humans linked an increase in blood serum of the glycerophospholipid lysophosphatidylcholine (LPC 16:0) to a high level of BAT activity when subjects were exposed to a short cold challenge. These authors suggested that the lipid increase observed could be correlated with the thermogenic mechanisms of BAT, and could stimulate the actions of UCP1, making it a possible biological predictor. Furthermore, all of these changes during adaptive thermogenesis can result in lipidome remodelling in BAT, which can actively regulate the metabolic processes that take place under a cold challenge [1,2]. Evidence of lipidomic changes in thermogenic BAT have been previously recorded in humans, mice, goats and pigs [2,3,17,18,19,20,21]. Hence, differences in lipidome composition of adipocytes may play a significant role in the rise of mitochondrial activity and thermogenic pathways, while enhancing energy communication with other tissues [1,3,22,23,24].

Previous studies [1,16] have utilised liquid chromatography–mass spectrometry (LC-MS)-based lipidomic analysis as tool to quantify a broad range of lipid species, in order to improve the understanding of the physiological role of lipids that were previously unknown or had no correlated function. However, information regarding the lipidomic profile of thermogenic BAT and the function behind many of its lipid players remains scarce. No studies were identified that quantified the differences of lipid species of BAT in new-born lambs when exposed to cold vs. ambient temperature. Therefore, the aims of this study were to identify the impact of short-term cold exposure (2 days) on new-born lamb BAT and plasma lipid composition, and to search for potential biomarkers to predict BAT activity through mass spectrometry analysis. 

## 2. Materials and Methods

This study was undertaken in spring 2018 at the Massey University Animal Physiology Unit (APU) with new-born Romney type lambs born on Keeble Farm (40°24′ S, 175°36′ E), Palmerston North, New Zealand. 

### 2.1. Animals and Sampling

During the lambing period, which was outdoors under pastoral conditions, fifteen new-born Romney lambs (9 males and 6 females) were randomly procured. Lambs were born as twins, but only one lamb per dam was used (the heaviest of the set). Within 12–24 h after parturition, the selected lamb was tagged and the ewe together with both of its lambs were brought indoors. The selected lamb within each twin set was then weighed and allocated randomly to one of the three treatment groups: Group 1 (*n* = 3; two males weighing 4.8 kg and 5.2 kg, respectively, and a female weighing 4.4 kg) as control, Group 2 (*n* = 6; two females weighing 4.9 and 4.5 kg, respectively, and four males weighing 4.6, 6.0, 4.6 and 5.3 kg, respectively) that were kept indoors for two days at an ambient temperature (20–22 °C) and Group 3 (*n* = 6; two females weighing 5.5 and 6.1 kg, respectively, and four males weighing 5.4, 4.5, 5.4 and 5.2 kg, respectively) that were kept indoors for two days at a cold temperature (4 °C). This followed the previously described method of Marcher et al. [2] to induce a cold challenge. Soon after being brought to the APU, all lambs in Group 1 were euthanised via captive bolt to provide a baseline lipidomic profile. Samples of brown adipose tissue (BAT) from around the kidneys were collected and stored at −80 °C. Lambs in Groups 2 and 3, together with their dams and siblings, were moved into indoor pens (2 m by 1 m) for two days. Blood samples (5 mL) from the jugular vein using a 22 G vacutainer needle from the lambs in Group 2 and 3 were collected into lithium heparinised tubes on days 0, 1 and 2. All blood tubes were centrifuged at 2000× *g* at 4 °C for 15 min, where the plasma was after aliquoted into 2 mL tubes and stored at −80 °C. Siblings of the lambs in Group 3, that were not subjects of the current study, were wrapped with wool covers (Woolover Limited, Christchurch, New Zealand) to enhance their comfort and to minimise the impact of the cold. During the two days of indoor retention, the ewes were fed commercial roughage (FiberEzy, Fiber Fresh Feeds Ltd., Reporoa, New Zealand) and commercial grain-based pellets (10%) (NRM Sheep Nuts, Northern Roller Mills, Christchurch, New Zealand) and had unrestricted access to water. The ewes and lambs were monitored at least 3 times per day during this period, to ensure successful ewe/lamb bonding and that the lambs were successfully suckling the ewe. On day 2, after 48 h exposure at respective temperatures, the 12 lambs of these two groups (i.e., 6 per group, in Groups 2 and 3) were weighed and euthanised by captive bolt. Samples of brown adipose tissue from around the kidneys were collected and stored at −80 °C. In all groups after the study lambs had been euthanised, their dams and remaining siblings were returned to Keeble Farm and to commercial farming conditions. 

### 2.2. Sample Preparation and Randomisation

All BAT and plasma samples were submitted to the Metabolomics Platform at AgResearch (Lincoln Research Centre, Lincoln, New Zealand) for mass spectrometry analysis of lipids. Plasma samples were stored at −80 °C prior to analysis. A two-step randomisation batch was created using the random number function in Microsoft Excel, to account for the repeated measurements from each lamb, while minimising the potential effect of analytical instrument drift on the results (e.g., a sensitivity may be lower at the end of an analytical batch compared to the start). Therefore, in the first randomisation step the order of the lambs in the analytical batch was randomised, and within the second randomisation step the order of each repeated measurement (i.e., 0, 1, 2 days) within each individual lamb was randomised. 

### 2.3. Extraction of Lipids from Brown Adipose Tissue

Brown adipose tissue was weighed and cut into 24 pieces while still frozen in a 4 × 6 matrix. Two grams of brown adipose tissue was placed into a centrifuge tube and placed at −80 °C for 1 h to refreeze the tissue. Forty milliliters of cold isopropanol (−20 °C) was added to the tube and the tissue was homogenised on ice using an Ultra Turrax homogeniser (T 25, IKA GmbH, Staufen, Germany). Ten microliters of the resulting brown adipose tissue slurry was added to a 2 mL microcentrifuge tube, along with 10 µL of an internal standard (Lipidomix Splash mix, Avanti Polar Lipids, Inc., Alabaster, AL, USA) and 1500 µL of butanol:methanol (1:1, *v*/*v*). The microcentrifuge tube was shaken in a bead shaker (Qiagen, Hilden, Germany) at 30 Hz for 5 min, and then sonicated for 1 h. The tube was then centrifuged at 16,000× *g* at room temperature for 10 min. The supernatant was then added to an amber chromatography vial for analysis.

### 2.4. Extraction of Lipids from Plasma

Plasma lipids were extracted using the method described by Huynh et al. [25]. Briefly, plasma samples were thawed at room temperature in the dark, mixed and 10 µL aliquoted into a 2 mL Eppendorf tube. Butanol/methanol with 5 mM ammonium formate (1:1, 95 µL) was added, along with 5 µL of an internal standard. Tubes were closed and vortexed in a multitube vortex mixer for 3 min at 2500 revolutions per minute at room temperature. The samples were then sonicated for 60 min and centrifuged at 16,000× *g* for 10 min at room temperature. The supernatant was transferred to amber chromatography vials prior analysis.

### 2.5. Lipidomic Analysis by LC-MS

Detection of lipids in BAT and plasma was carried out using a Shimadzu LCMS 9030 LC-qTOF mass spectrometer. Separation was carried out through a Waters Acquity CSH C18 column (1.7 µm particle size, 2.1 × 100 mm ID) at 65 °C and eluted over a 17 min gradient with a flow rate of 400 μL min^−1^. Mobile phase A was water:acetonitrile:isopropanol (50:30:20) with 20 mM ammonium formate, and mobile phase B was water:acetonitrile:isopropanol (1:9:90) with 20 mM ammonium formate. The gradient elution programme was as follows: 10–45% B (0–2.7 min), 45–53% B (2.7–2.8 min), 53–65% B (2.8–9 min), 65–89% B (9–9.1 min), 89–92% B (9.1–11 min), 92–100% B (11–11.1 min), held at 100% B (11.1–13.9 min), 100–10% (13.9–14 min), held at 10% B (14–17 min) [26]. The autosampler was held at 20 °C. Two microliters of plasma extract and 0.5 µL of BAT extract were injected onto the column. The mass spectrometer acquired data in “full scan” mode between *m*/*z* 50 and 1200, and in data independent acquisition MS/MS mode across the same range in 20 *m*/*z* windows to acquire fragmentation data on all lipids, with a collision energy ramp from 6 to 23 eV. Loop time for the MS method was 0.5 s. 

### 2.6. Post-Processing and Analysis of Lipidomic Data

Data were converted into the mzML format and processed using MS DIAL [27]. BAT lipidomic data were normalised firstly by the weight of the tissue extracted. Afterwards, all data, BAT and plasma, were normalised based on the LOWESS algorithm in MS DIAL and exported for manual curation (checking for duplicate identifications, merging adducts with the same identification and data quality checking). Identification (lipid ontology, category, main and sub-class) was based on matching against the Lipidmaps database [28] in MS DIAL. 

### 2.7. Statistical Analysis

Statistical analyses were only undertaken for lipids with MS2 level identification and known ontology and lipid names. An average of the three BAT baseline samples from the control lambs (Group 1) was calculated. This average was used to correct all BAT lipidomics results from the lambs in Groups 2 and 3, by subtracting its value from each and all treatment values. Individual lamb plasma data recorded at day 1 and 2 (24 h and 48 h exposure, respectively) were baseline corrected with respective recordings of day 0. This baseline correction was carried out by subtracting the value recorded at day 0 to the correspondent values recorded at day 1 and day 2, for each sample accordingly. The basis behind this approach, instead of using the value of day 0 as a covariate, was because the statistical analysis was undertaken through the LIPID MAPS^®^ online tool (www.lipidmaps.org/resources/tools/stats, accessed on 17 February 2022) [29], which does not have a covariate option to use control BAT samples or plasma samples of day 0 to analyse the treatment samples. For that reason, the baseline corrected BAT and plasma lipidomic data were used for LIPID MAPS^®^ analysis. 

An analysis of variance (ANOVA) was utilised for the comparison between samples from ambient temperature lambs vs. those cold exposed (i.e., Group 2 vs. Group 3, respectively), with a *p*-value cutoff of 0.05. In addition, an orthogonal projection to latent structure discriminant analysis (OPLS-DA) was constructed via LIPID MAPS^®^ to visualise the differences between cold vs. ambient temperature exposed lambs for: BAT, plasma day 1 and plasma day 2 samples. Plots of lipid class figures for BAT and plasma (days 1 and 2 of exposure) were constructed in R (version 4.1.0) [30], inside RStudio (version 1.10.0) [31] with the “tidyverse” package (version 1.3.1) [32], the “ggplot” package (version 3.3.5) [33], the “ggnewscale” package (version 0.4.7) [34] and the “RColorBrewer” package (version 1.1.2) [35]. 

## 3. Results

**Figure 1:**
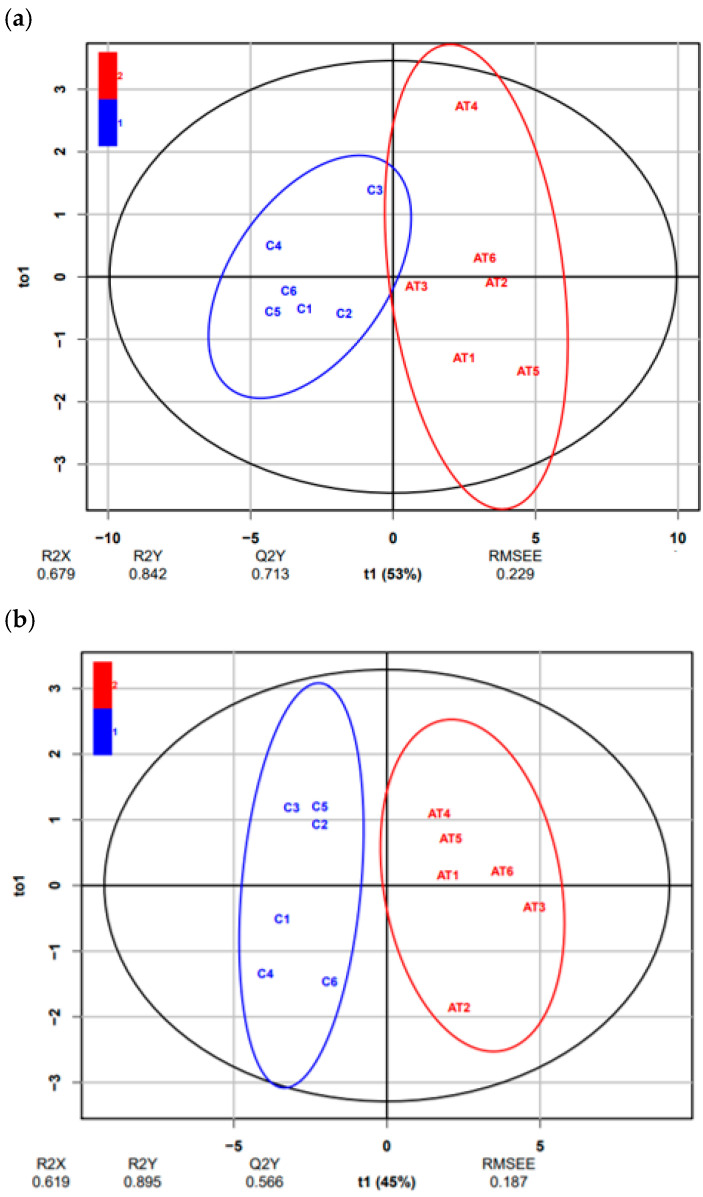

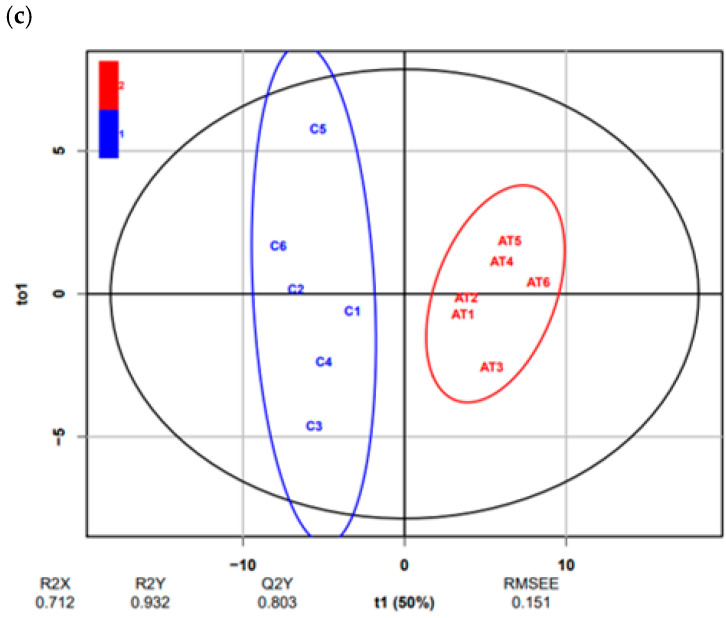
OPLS-DA score plot of (**a**) 20 different lipids (*p*-value < 0.05), between brown adipose tissue (BAT) samples from Group 2 (20–22 °C, AT in red) vs. Group 3 (4 °C, cold in blue), after 2 days of exposure; (**b**) 20 different lipids (*p*-value < 0.05), between plasma samples from Group 2 (20–22 °C, AT in red) vs. Group 3 (4 °C, cold in blue), after 1 day of exposure; (**c**) 74 different lipids (*p*-value < 0.05), between plasma samples from Group 2 (20–22 °C, AT in red) vs. Group 3 (4 °C, cold in blue), after 2 days of exposure.

A total of 1438 unique known lipids were found from LC-MS analysis within all BAT and plasma samples taken together from days 1 and 2 after exposure, with 101 being significantly different (*p* < 0.05) between samples collected from ambient temperature conditions (Group 2) vs. samples from cold conditions (Group 3) (Table S1). The 101 significantly different lipids were categorised as: glycerolipids (GLs), glycerophospholipids (GPs), sphingolipids (SPs) and sterol lipids (STs). All sub-classes included within these categories and their abbreviations are described in Table 1. These 101 significantly different lipids contributed the most to the separation observed in the OPLS-DA plot for BAT and plasma (days 1 and 2, after exposure), and between ambient temperature (Group 2) vs. cold conditions (Group 3) clusters (Figure 1).

In each plot, the horizontal component (X-axis) of the OPLS-DA score depicts the variation between the groups and the vertical component (Y-axis) depicts the variation within the groups. Class ellipses represent the 95% confidence regions for each group. R2X, variation of X-axis that is explained by the model; R2Y, variation of Y-axis that is explained by the model; Q2Y, goodness of model prediction; t01, first orthogonal component; t1, first principal component; RMSEE, root mean square error of estimation.

### 3.1. Brown Adipose Tissue (BAT)

**Figure 2:**
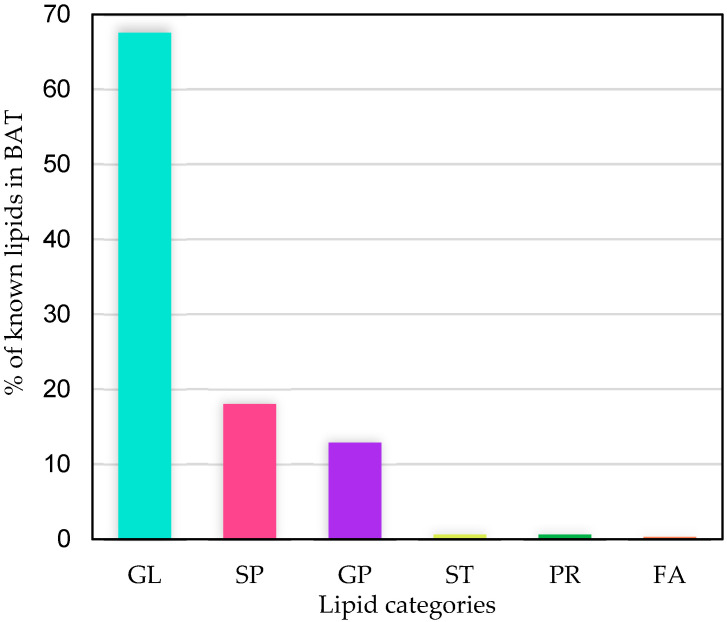
Percentages of the different categories of found known lipids in all lamb brown adipose tissue (BAT) samples. GL, glycerolipids; SP, sphingolipids; GP, glycerophospholipids; ST, sterol lipids; PR, prenol lipids; FA, fatty acyls.

A total of 291 unique known lipids were found from LC-MS analysis of BAT samples. The majority of lipids were glycerolipids (191 lipids, 67.52% of total), with the remainder being sphingolipids (56 lipids, 18.01% of total), glycerophospholipids (39 lipids, 12.86% of total), sterol lipids (2 lipids, 0.64% of total), prenol lipids (2 lipids, 0.64% of total) and fatty acyls (1 lipid, 0.32% of total) (Figure 2).

**Figure 3:**
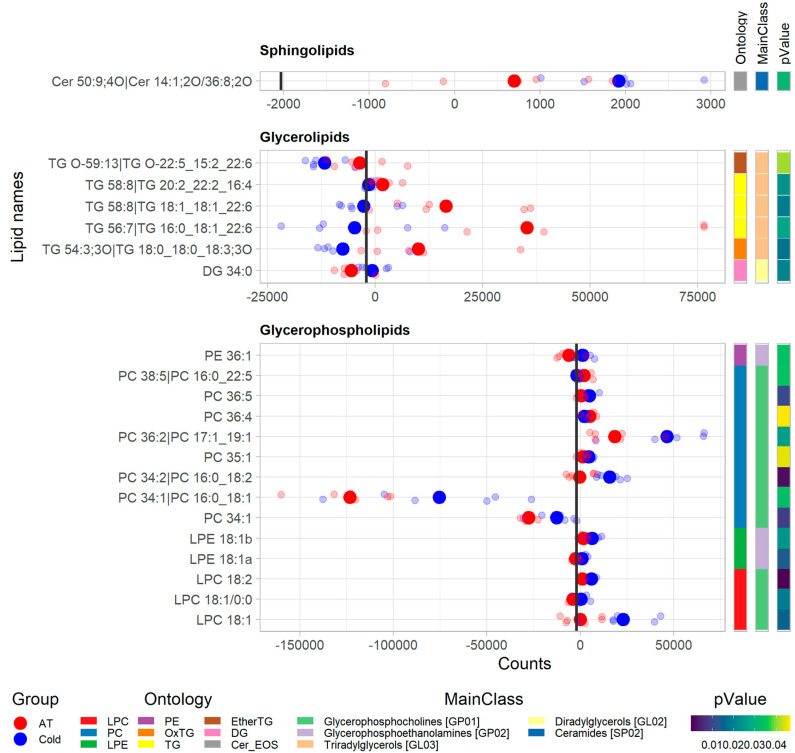
Plots of significantly different (*p* < 0.05) lipid classes within lipid categories in brown adipose tissue (BAT) samples from new-born lambs kept at ambient temperature (20–22 °C, AT in red) vs. those kept at cold temperature (4 °C, cold in blue) after 2 days of exposure. Each row of dots represents a lipid metabolite with respect to its ion counts obtained from the LC-MS analysis, where big dots represent the mean and smaller dots represent the individual values (*n* = 6 in each group). The vertical bold black line represents the average count value across all BAT datasets.

Of the 291 unique known lipids detected in the BAT samples, 20 were significantly different (*p*-value < 0.05) between ambient temperature conditions (Group 2) vs. cold conditions (Group 3) (Figure 3). The majority of these 20 lipids comprised glycerophospholipids (13), followed by glycerolipids (6) and sphingolipids (1). Under cold conditions (Group 3), the sphingolipid Cer 50:9;4O|Cer 14:1;2O/36:8;2O and most of the glycerophospholipids in BAT were more abundant (in terms of ionic counts) compared with that in the ambient temperature conditions (Group 2). However, most glycerolipids and two glycerophospholipids (PC 36:4 and PC 38:5|PC 16:0_22:5) were scarcer under cold conditions, compared with the ambient temperature conditions.

### 3.2. Plasma

**Figure 4:**
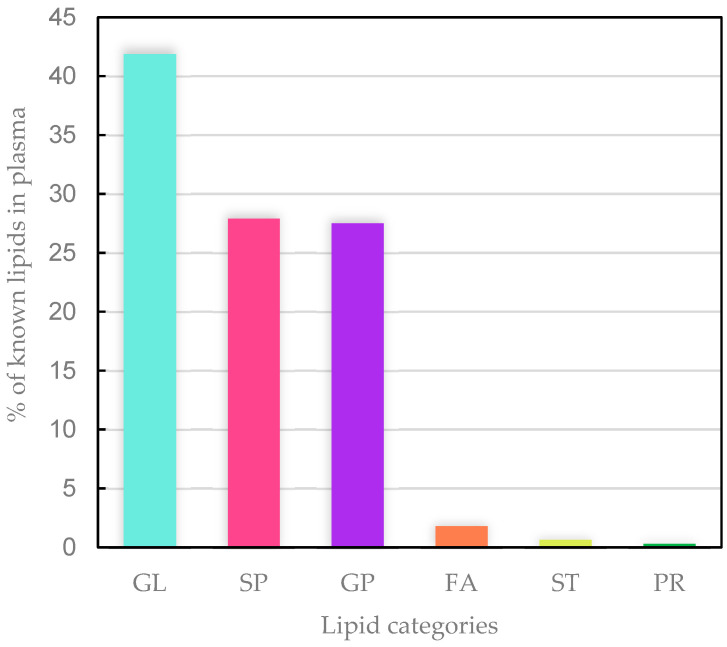
Percentages of the different categories of found known lipids in all lamb plasma samples. GL, glycerolipids; SP, sphingolipids; GP, glycerophospholipids; FA, fatty acyls; ST, sterol lipids; PR, prenol lipids.

A total of 1147 unique known lipids were found from LC-MS analysis of plasma samples. The majority of the known lipids were glycerolipids (498 lipids, 41.88% of total), with the remainder being sphingolipids (331 lipids, 27.89% of total), glycerophospholipids (286 lipids, 27.50% of total), fatty acyls (20 lipids, 1.80% of total), sterol lipids (8 lipids, 0.63% of total) and prenol lipids (4 lipids, 0.31% of total) (Figure 4). 

**Figure 5:**
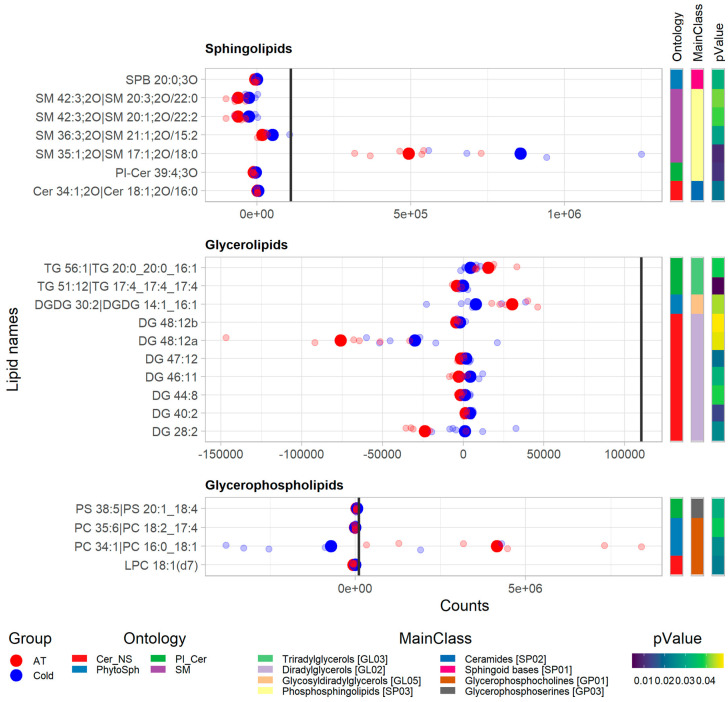
Plots of significantly different (*p* < 0.05) lipid classes within lipid categories in plasma samples from new-born lambs kept at ambient temperature (20–22 °C, AT in red) vs. those kept at cold temperature (4 °C, cold in blue), after 1 day of exposure. Each row of dots represents a lipid metabolite with respect to its ion counts obtained from LC-MS analysis, where the big dots represent the mean and smaller dots represent individual values (*n* = 6 in each group). The vertical bold black line represents the average count value across all plasma datasets, after 1 day of exposure.

Of the 1147 unique known lipids found in the plasma samples, 20 were significantly different (*p* < 0.05) between ambient temperature conditions (Group 2) vs. cold conditions (Group 3), after one day of exposure at the respective temperatures (Figure 5). Of these 20 lipids, most were glycerolipids (9), followed by sphingolipids (7) and glycerophospholipids (4). In the plasma of lambs under cold conditions (Group 3), after 1 day of exposure, all sphingolipids recorded and most glycerolipids and glycerophospholipids were increased when compared with the plasma samples from the lambs kept at ambient temperature (Group 2). However, two specific glycerolipids (TG 56:1|TG 20:0_20:0_16:1 and DGDG 30:2|DGDG 14:1_16:1) and one glycerophospholipid (PC 34:1|PC 16:0_18:1) were decreased when compared with the plasma samples under ambient temperature conditions (Group 2).

**Figure 6:**
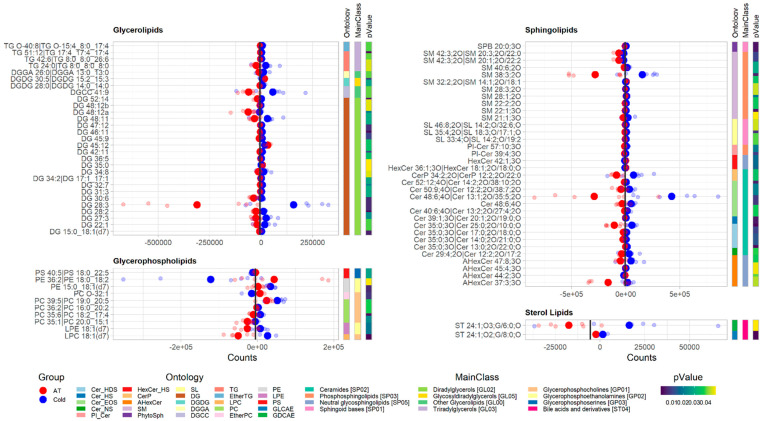
Plots of significantly different (*p* < 0.05) lipid classes within lipid categories in plasma samples from new-born lambs kept at ambient temperature (20–22 °C, AT in red) vs. those kept at cold temperature (4 °C, cold in blue), after 2 days of exposure. Each row of dots represents a lipid metabolite with respect to its ion counts obtained from LC-MS analysis, where big dots represent the mean and smaller dots represent the individual values (*n* = 6 in each group). The vertical bold black line represents the average count value across all plasma datasets, after 2 days of exposure.

Of the 1147 unique known lipids detected in plasma samples, 74 were significantly different (*p*-value < 0.05) between the ambient temperature (Group 2) and cold (Group 3) conditions, after 2 days of exposure of lambs at the respective temperatures. The majority of the 74 lipids comprised sphingolipids (34 lipids), followed by glycerolipids (28 lipids), glycerophospholipids (10 lipids) and sterol lipids (2 lipids) (Figure 6). In plasma, under cold conditions, all sterol lipids and most sphingolipids, glycerolipids and glycerophospholipids were more abundant than in the plasma samples under ambient temperature conditions. However, four specific sphingolipids (HexCer 36:1;3O|HexCer 18:1;2O/18:0;O, HexCer 42:1;3O, AHexCer 44:2;3O and SM 32:2;2O|SM 14:1;2O/18:1), three glycerolipids (DG 45:12, DG 35:0 and DGDG 30:5|DGDG 15:2_15:3) and three glycerophospholipids (PC O-32:1, PE 36:2|PE 18:0_18:2 and PS 40:5|PS 18:0_22:5) were less prevalent, compared with those in plasma samples under ambient temperature conditions. 

## 4. Discussion

Exposure to cold leads to a significant cascade of events, where lipidome remodelling can influence and regulate adipocyte function towards thermogenesis [18]. In the present study, lipid profiles of BAT and plasma were generated through LC-MS analysis, with the aim of determining the lipidomic changes that occur in new-born lambs exposed to cold conditions. The results, even with a relatively small number of lambs, demonstrated that this short cold challenge (2 days) induced significant changes in BAT and the plasma lipidome, by increasing the abundance of many of the lipid classes found.

Under the cold conditions of the present study, glycerolipids such as DGs were increased in plasma, and were more abundant over time, compared with the ambient temperature conditions. This increased abundance could be associated with lipid metabolism and TAG turnover [36], which seems to be connected with the decreased TAGs seen in BAT under the cold conditions. These results are consistent with previous studies, where the most marked changes in the BAT lipidome under cold conditions were related to the hydrolysis of TGs into DGs [18,37,38]. Therefore, this decline of TAGs in BAT could indicate that a hydrolytic action by lipases was occurring [39,40], resulting in the release of fatty acids. According to Bartelt et al. [38], TAGs were decreased in activated BAT of mice under cold exposure, and TAG turnover was accelerated in plasma. This observation can be correlated to the abundance of diacylglycerols seen in plasma in the present study, which increased with time during the cold challenge. This lipolytic action of TAGs is triggered by the sympathetic stimulation of β-adrenergic receptors, induced by cold exposure [41,42,43], which is required in order to produce free fatty acids for the thermogenic activation of BAT [44,45]. Moreover, mitochondrial activity becomes supported under the thermogenic adaptation and the released free fatty acids are used in BAT as the substrate for uncoupled oxidation by UCP1, thus generating heat [1,7].

The two types of sterol lipids detected, GLCAE and GDCAE, were observed to be greater in plasma after 2 days of cold exposure. These particular types of lipids correspond to a bile acid class. It is known that bile acids can regulate adipocyte physiology by taking part in lipolysis [46], and in BAT they can induce thermogenesis [47]. Sterol lipids can also affect membrane fluidity and permeability [48], therefore the increase seen in these lipids could be boosting the lipolytic actions on TAGs as well as channeling the posterior free fatty acid uptake into BAT. In addition, all of the glycerolipids found to be significantly different in abundance between the cold vs. ambient environments contained very-long-chain fatty acyls. Previous studies with short-term cold challenges have associated an enrichment of very long acyl chains with TAGs [2], glycerophospholipids and sphingolipids in the adipose tissue [37]. The above-mentioned studies suggest that these lipid types could be functionally involved during thermogenic adaptation, as it is known that long-chain fatty acyls can activate UCP1 more efficiently [49]. Accordingly, not only did all glycerolipids, but all glycerophospholipids and sphingolipids, recorded and analysed here corresponded to very-long-chain fatty acyls. 

Marked glycerophospholipid remodelling was observed during the 2-day exposure to cold, in BAT and plasma. The abundance of glycerophospholipids has been recorded in BAT [18,36], and reported to increase under cold exposure [37]. Here, several lipid classes such as PC, LPC, PE and LPE were increased in the present study under cold conditions, compared with ambient temperature conditions. These observations are similar to the ones stated by Marcher et al. [2], where short-term cold exposure in mice induced an increase in these lipid classes in BAT. This increase could be correlated with a response and adaptation to cold exposure by having significant functional implications. Accordingly, Hoene et al. [50] reported that there is a great abundance of phospholipids in heat-generating BAT that could have been correlated to a high density of mitochondria, since mitochondrial fission can increase its density within an hour when a big source of energy/heat is needed [51]. Additionally, phospholipid metabolism has been reported to be activated during both short and long cold exposure in mice [2,19], where it may have a supportive role for mitochondrial biogenesis. In addition, differences in the composition of acyl chains in the total mitochondrial phospholipids in BAT were previously observed, while adrenergic stimulation was occurring under the influence of long-term cold exposure [52]. Therefore, considering these previous findings, the elevated content of glycerophospholipids seen in the current study could suggest that there is an increased mitochondrial content and function as a response to cold exposure, leading to an increase in the thermogenesis mechanisms. 

As mentioned previously, all lipid categories that were significantly increased after cold exposure contained very-long-chain fatty acyls. Schweizer et al. [53] reported that thermogenic adipocytes had higher contents of PC and PE constituted by unsaturated long chains of approximately 36 carbons. Similar observations of these lipid classes were seen in an adipose tissue comparison in mice, where a higher abundance of unsaturated long-chain PC and PE was found in BAT rather than in white adipose tissue (WAT) [17]. It is known that glycerophospholipids are major components of cellular membranes, regulating fluidity, homeostasis and dynamics [54], where PC and PE are key components that could determine the fate of adipocyte function and structure [16]. Consequently, this increase in unsaturated long-chain glycerophospholipids might have an influence on the membrane fluidity, particularly in mitochondria, thereby enhancing BAT uptake of the free fatty acids available through TAG lipolysis. 

An indication of BAT activity that is correlated with cold-induced thermogenesis can be inferred by glycerophospholipid metabolites such as lysophophatidylcholines [16]. Previously, Boon et al. [17] associated LPC 16:0 with BAT activity in humans that were exposed to cold, stating that the increase in this lipid could stimulate the actions of UCP1. In the current study, most LPCs and PCs increased in cold conditions both in BAT and plasma. Specifically, LPC 18:1 increased its mean value 11 times in plasma from 1 day compared with 2 days of exposure, similarly PC 35:6 increased twice in the same time span. These results may suggest that this significant increase in both metabolites in plasma could be associated with a response to cold exposure seen through time, and therefore, generated as a consequence of BAT activity. Specifically, PC 35:6 and, more importantly, LPC 18:1, could serve as potential markers to predict BAT activity in a non-invasive way, since there are no methods known to the authors to either quantify the amount or the activity of BAT in lambs. Further studies would be needed to validate these biomarkers in plasma for BAT metabolic activity, which could be used to provide an indirect estimate of BAT activity and reserves in new-born lambs. If validated, they could contribute to further research and animal selection focused on improvement of new-born lamb survival under cold conditions. 

In contrast to glycerophospholipids and glycerolipids, sphingolipids are not usually abundant in the body [55]. Nonetheless, a significant increase in many classes of sphingolipids was observed in BAT and plasma after cold exposure. These types of lipids are fundamental for cell proliferation [56]. They are mainly found in plasma membranes [57], where they are involved in cellular transport and signal transduction [58,59]. Within this category, SMs are one of the biggest components of plasma membranes [60], where they can be further metabolised into ceramides [61]. Together with the actions of SM synthase, ceramides can utilise PC in order to increase the levels of SM and DGs [60]. It may be the case that these co-processes were occurring in the study animals, where not only SM, but several classes of ceramides were seen jointly increased in the plasma of cold-exposed lambs. Moreover, as PC levels increased when exposed to cold, it may be that the SM synthase machinery was indeed active, inducing the increment of both SMs and DGs. As discussed above, DG levels were observed to be significantly increased in BAT and plasma after cold exposure in the present study. Therefore, it could be suggested that these observations had a correlation with this secondary result from sphingolipid biosynthesis, thus increasing DGs. Besides having a role as a major hub of sphingolipid metabolism [1], ceramides have an important physicochemical and structural function in cell membranes. According to Alexaki et al. [59], sphingolipid metabolites and ceramides are bioactive and can change the cell activity by interaction between intracellular targets and cell-surface receptors. Sphingolipids and cholesterols compose up to 30% of the plasma membrane surface of adipocytes, which confers these cells with a detergent-resistant characteristic [62,63]. This ability to prevent detergent elements from compromising the cell structure and function seems to be essential. The main function of the adipocytes is to take up and release fatty acids, which are known to be mild detergents [59]. In response to cold exposure, there will be an enhanced transport and uptake of fatty acids to BAT, as the basic substrate to thermogenesis. Therefore, it is imperative that the cell can manage this entire metabolic function. For that reason, Meshulam et al. [63] implied that this detergent-resistant configuration of sphingolipids may protect the adipocyte membrane from deleterious effects when they are managing high concentrations of fatty acids, for transport or metabolism. Moreover, a previous study demonstrated that de novo sphingolipid biosynthesis is indispensable for adipocyte cell viability and normal metabolic function [59]. A subsequent study on cold-induced inguinal WAT in mice [37] linked cold exposure to the activation of de novo sphingolipid synthesis, as an increase in sphingomyelin and ceramides. These authors reasoned that this increase in sphingolipids was a consequence of the elevated availability of free fatty acids that were induced by cold exposure. In addition, free fatty acids may trigger ceramide synthesis [64,65]. Therefore, the present results can be paired up with these previous observations, where the increase in many sphingolipid classes such as ceramides and SMs observed in BAT could have had profound beneficial effects for the thermogenic adipocytes. This increase could provide a higher protection to the adipocyte’s integrity, while still being able to utilise fatty acids as a means to produce heat when exposed to cold.

## 5. Conclusions

Collectively, these data demonstrate that in new-born lambs, short-term cold exposure (2 days) induces profound changes in BAT and the plasma lipidome. Significant increases in lipid composition of glycerolipids, glycerophospholipids, sphingolipids and sterol lipids seem to cooperate as one, in order to enhance lipid metabolism via BAT thermogenic activation and adipocyte survival during cold adaptation. Experiments which further analyse the roles of these lipid changes and validate potential biological markers for BAT activity, such as LPC 18:1 and PC 35:6, will provide additional insights into the mechanisms involved during cold adaptation and further contribute to the improvement of new-born lamb survival under cold conditions.

## Figures and Tables

**Table 1:** Abbreviations of lipid sub-classes used throughout this paper.

Category	Lipid Sub-Class	Abbrev.
Glycerolipids	Triacylglycerol	TG
	Ether-linked triacylglycerol	EtherTG
	Oxidised triglyceride	OxTG
	Diacylglycerol	DG
	Digalactosyldiacylglycerol	DGDG
	Diacylglyceryl-3-O-carboxyhydroxymethylcholine	DGCC
	Diacylglyceryl glucuronide	DGGA
Glycerophospholipids	Lysophophatidylcholine	LPC
	Phosphatidylcholine	PC
	Ether-linked phosphatidylcholine	EtherPC
	Lysophosphatidylethanolamine	LPE
	Phosphatidylethanolamine	PE
	Phosphatidylserine	PS
Sphingolipids	Sphingomyelin	SM
	Sulfonolipid	SL
	Phytosphingosine	SPB
	Ceramide	Cer
	Oxidised ceramide phosphoinositol	PI_Cer
	Ceramide hydroxy fatty acid-sphingosine	Cer_HS
	Ceramide hydroxy fatty acid-dihydrosphingosine	Cer_HDS
	Ceramide Esterified omega-hydroxy fatty acid-sphingosine	Cer_EOS
	Ceramide non-hydroxyfatty acid-sphingosine	Cer_NS
	Ceramide 1-phosphates	CerP
	Hexosylceramide hydroxyfatty acid-sphingosine	HexCer
	Acylhexosylceramide	AHexCer
	Hexosylceramide hydroxyfatty acid-sphingosine	HexCer_HS
	Phytosphingosine	PhytoSph
Sterol Lipids	Esterified glycodeoxycholic acid	GDCAE
	Esterified glycolithocholic acid	GLCAE

## Data Availability

Not applicable.

## Supplementary Materials

The following supporting information can be downloaded at: https://www.mdpi.com/article/10.3390/ani12202762/s1, Table S1. Lipidomics data of BAT and plasma after 1 and 2 days of cold exposure.
